# An Innovative Digestion Method: Ultrasound-Assisted Electrochemical Oxidation for the Onsite Extraction of Heavy Metal Elements in Dairy Farm Slurry

**DOI:** 10.3390/ma14164562

**Published:** 2021-08-13

**Authors:** Chenyu Li, Bin Xue, Shang Wang, Xi Zhang, Chen Zhao, Xiaobo Yang, Run Zhao, Lin Dai, Shengqi Su, Haoqi Xu, Zhiqiang Shen, Zhigang Qiu, Jingfeng Wang

**Affiliations:** 1 Department of Environment and Health, Tianjin Institude of Environmental and Operational Medicine, Tianjin 300050, China; nk_lcy710430@hotmail.com (C.L.); xue_bin04@163.com (B.X.); wsh847@163.com (S.W.); Zhangxi0820@126.com (X.Z.); zhaochen212@126.com (C.Z.); 18072712080@163.com (X.Y.); ssqsuqi@163.com (S.S.); Haoqi_xu@126.com (H.X.); tianjinszq922@sohu.com (Z.S.); 2 Agro-Environmental Protection Institute, Ministry of Agriculture and Rural Affairs, Tianjin 300191, China; 15900389657@163.com; 3 Tianjin Key Laboratory of Pulp and Paper, Tianjin University of Science and Technology, Tianjin 300457, China; dailin@tust.edu.cn

**Keywords:** biomass resource, dairy slurry, digestion efficient, heavy metals, ultrasound-assisted electrochemical oxidation

## Abstract

Dairy farm slurry is an important biomass resource that can be used as a fertilizer and in energy utilization and chemical production. This study aimed to establish an innovative ultrasound-assisted electrochemical oxidation (UAEO) digestion method for the rapid and onsite analysis of the heavy metal (HM) contamination level of dairy slurry. The effects of UAEO operating parameters on digestion efficiency were tested based on Cu and Zn concentrations in a dairy slurry sample. The results showed that Cu and Zn digestion efficiency was (96.8 ± 2.6) and (98.5 ± 2.9)%, respectively, with the optimal UAEO operating parameters (digestion time: 45 min; ultrasonic power: 400 W; NaCl concentration: 10 g/L). The digestion recovery rate experiments were then operated with spiked samples to verify the digestion effect on broad-spectrum HMs. When the digestion time reached 45 min, all digestion recovery rates exceeded 90%. Meanwhile, free chlorine concentration, particle size distribution, and micromorphology were investigated to demonstrate the digestion mechanism. It was found that 414 mg/L free chorine had theoretically enough oxidative ability, and the ultrasound intervention could deal with the blocky undissolved particles attributed to its crushing capacity. The results of particle size distribution showed that the total volume and bulky particle proportion had an obvious decline. The micromorphology demonstrated that the ultrasound intervention fragmented the bulky particles, and electrochemical oxidation made irregular blocky structures form arc edge and cellular structures. The aforementioned results indicated that UAEO was a novel and efficient method. It was fast and convenient. Additionally, it ensured digestion efficiency and thus had a good application prospect.

## 1. Introduction

Dairy farm slurry is an important, cheap biomass resource [1,2,3] rich in mineral nutrients and lignocellulose. With the rapid increase in the number of large-scale livestock farms in the last few years in China, plenty of livestock feces and slurry are discharged into the nearby natural environment, resulting in ecological pressure [4,5]. The resource utilization methods of dairy slurry include returning cropland as a fertilizer [6,7], energy engineering [8], and chemical production [9,10]. Practical experience indicates that returning cropland is the most practical and common treatment approach for farm slurry because it is rich in nutrients (N, P, and K) [11]. Unfortunately, the high abundance of heavy metals (HMs) may result in a serious ecological hazard. The HMs in farm slurry can be fixed in the soil along with returning cropland, keeping an average concentration of several mg/kg to tens of mg/kg for a long time [12]. HM pollution consistently threatens environmental ecology due to its toxic, accumulative, and persistent nature in the environment [5]. More seriously, the transfer of HMs from soil to cereals and plants is a major HM intake route for humans. Excessive HMs may accumulate in specific human organs and interact with proteins and enzymes, making them damaged or inactive [13].

Although the Chinese government has developed many management policies and treatment technologies to reduce the HM pollution from livestock slurry to environment, the lack of the onsite digestion-detection method is still the bottleneck to realize the effective control of the ecological risk due to HMs during returning cropland. Some novel detection methods have been developed for the onsite quantitative analysis of HMs in the last few years. Wen et al. established a portable tungsten coil electrothermal atomic absorption spectrometer for HM field analysis [14,15,16]. Wang et al. designed and implemented a field-based HM detection system involving electrochemical differential pulse anodic stripping voltammetry [17]. Additionally, with the advent of microfluidics technology, colorimetric sensors for the rapid detection of HMs can even work on a handheld device [18].

Compared with the detection methods, these recently reported digestion methods [19,20,21,22,23] were still challenging to implement in the field. In theory, the digestion procedure should ensure that treated samples are completely dissolved and HMs are released in a positive ion form compatible with the analytical method [24,25]. The conventional wet digestion procedures were relatively complicated and hazardous. Heavy use of strong acids (HClO_4_, HF, H_2_SO_4_, HCl, etc.) and heating devices restricted the digestion procedures to be operated in a normative chemical laboratory. Although some modified digestion methods have been recently established to reduce operation difficulty, the inevitable use of acids and laboratory devices (such as a microwave) still cannot meet the onsite digestion requirement.

Therefore, more safe and simple digestion methods need to be established to analyze the HM contamination level of livestock slurry. This study reported an innovative digestion method based on ultrasound-assisted electrochemical oxidation (UAEO) theory. The UAEO method was designed as a specialized technology for the dairy farm slurries on-site digestion, which had the potential to be applied in other farm slurry and sewage samples. The digestion extraction effects for major (Cu and Zn) and trace (Cr, Cd, Pb, Ba, Co, Ni, Bi, and Ag) HMs were proved with the livestock slurry samples. Furthermore, the digestion mechanism of the reported method was analyzed and discussed to elaborate the functioning process.

## 2. Materials and Method

### 2.1. Reagents, Standards, and Samples

All reagents used in this study were at least analytical grade. All solutions were prepared in deionized water (resistivity ≥18.2 MΩ·cm). A commercially multi-element standard solution (Thermo Scientific Co. Ltd., Waltham, MA, USA) was used to analyze the standard digestion rate.

**Figure 1:**
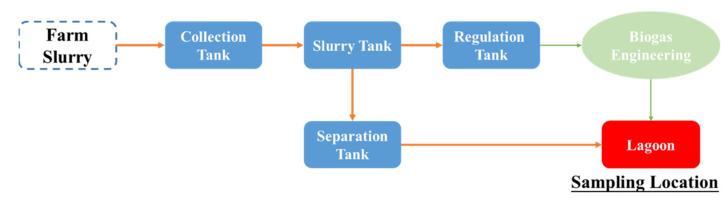
Fecal slurry process flow in three large-scale dairy farms.

The dairy farm slurry samples were collected from three typical large-scale crop–animal mixed farms in July 2019 in Tianjin of China. All breeding varieties were Chinese Holstein Cattle. The cattle breeding stock of every farm was above 1000 units. The fecal slurry process flow in three large-scale dairy farms is shown in Figure 1. One of the dairy farms employed biogas engineering as a recycling treatment technique, while the other two carried out only a sedimentation liquid–solid separation. In any case, the lagoon was the unique storage facility before returning cropland. Therefore, all slurry samples were collected from the lagoon in experiments.

The slurry equally sampled from three random points in a lagoon was uniformly mixed and stored in a clean-washed polyethylene plastic bottle. All samples were filtered with 10-mesh sieves to remove macroparticle impurities.

### 2.2. Construction of the UAEO Digestion Apparatus

**Figure 2:**
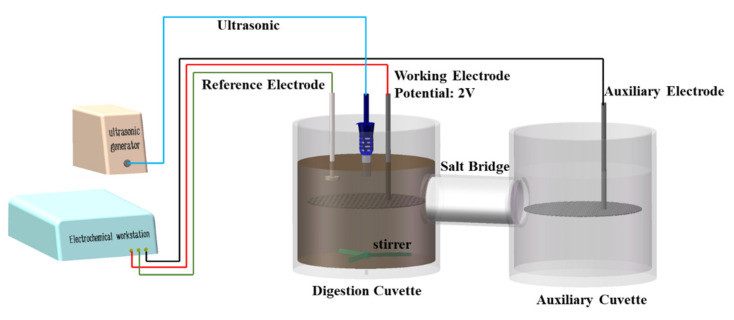
Schematic diagram of the UAEO digestion apparatus.

As shown in Figure 2, the UAEO digestion apparatus was mainly constructed using two cuvettes connected with a NaCl salt bridge. The volume of both cuvettes was approximately 120 mL. The ingredients of the salt bridge solution were 20% (*w*/*v*) NaCl and 2% (*w*/*v*) agar. The aforementioned solution was heated to a boil, infused into the connected part, and cooled down to form a gel. The electrochemical oxidation function was implemented using a classic three-electrode system. The working and auxiliary electrodes were made using Ru–Ir-coated titanium ((Ru–Ir)@Ti) inert metal material, while the reference electrode was an Ag–AgCl electrode. The working potential was controlled at 2 V using a CHI-760D electrochemical workstation (Science Days Technology Co. Ltd., Beijing, China). The ultrasound function was implemented using a Scientz-IID ultrasonic generator (Scientz Co. Ltd., Ningbo, China), while the ultrasonic duty ratio was set to 50% (5 s/5 s). In the digestion cuvette, a stirrer was installed at the bottom to blend the solution along with digestion.

### 2.3. UAEO Digestion Method and Zn and Cu Digestion Efficiency Test

To determine Cu and Zn digestion efficiency in slurry samples, the aqua regia with closed-vessel microwave digestion (AD) method was applied and compared with the UAEO digestion method. The AD method was performed as previously reported [26]. Briefly, 5.0 g of accurately weighed slurry sample, 10 mL of aqua regia (3:1, *v*/*v*, HCl:HNO_3_), and 5 mL of H_2_O_2_ (30%, *v*/*v*) were successively added in a poly tetra fluoroethylene (PTFE) vessel. The PTFE vessel was capped tightly and placed in a WX-6000 microwave apparatus (PreeKem Co. Ltd., Shanghai, China). The operating program of the microwave is shown in Table S1. After cooling down, the digestion solution was completely transferred and made up to 50 mL with deionized water.

The UAEO digestion method was operated with the following steps: (Ⅰ) 10.0 g slurry sample and a certain quality (testing range: 0.1–10 g) NaCl were accurately added into deionized water to make 100 mL. The aforementioned suspension was poured into the digestion cuvette. (Ⅱ) A NaCl solution with the same volume and concentration was poured into the auxiliary cuvette as in the digestion cuvette. (Ⅲ) The electrochemical workstation and the ultrasonic generator were started at the same time, sampling approximately 5 mL of digestion suspension in 0, 10, 20, 30, 45, and 60 min. The ultrasonic power was set in the test range of 0–600 W.

Both AD and UAEO samples were filtered to remove undissolved solid before HM analysis. The concentrations of Cu and Zn were tested using an AA-7000 atomic absorption spectrophotometer (Shimadzu Co. Ltd., Kyoto, Japan). The digestion efficiency was calculated using Equation (1).

Digestion efficiency = *C*_UAEO_/*C*_AD_ × 100% (1)

where *C*_UAEO_ is the concentration of the tested element digested by the UAEO method, while *C*_AD_ is that digested by the AD method.

### 2.4. Digestion Recovery Rate

The digestion recovery rate experiment was performed to monitor the UAEO method digestion effect for trace HMs. The slurry sample with the standard substance was prepared before the test. Specifically, 10 g slurry sample and 10 mL of multi-element standard solution were mixed accurately and shaken on an HNYC-203T constant-temperature shaking table (Honour Co. Ltd., Tianjin, China) for 6 h to form nonionic compounds (such as coordination compounds). A NaCl solution was used to increase the volume of the aforementioned sample to 100 mL. The UAEO method was executed with the optimal NaCl concentration and ultrasonic power verified by the digestion efficiency result. The digestion suspension was sampled for 0, 20, and 45 min to analyze the standard digestion rate.

The concentrations of 10 objective HMs (Zn, Cu, Cr, Cd, Pb, Ba, Co, Ni, Bi, and Ag) were tested using a Thermo 7400 Inductively Coupled Plasma Optical Emission Spectrometer (ICP-OES) (Thermo Scientific Co. Ltd., Waltham, MA, USA). The ICP-OES instrumental parameters for the analysis are listed in Table S2. The digestion recovery rate of every HMs was calculated using Equation (2).

Digestion recovery rate = (*C*_1_ − *C*_0_)/*C*_spk_ × 100% (2)

where *C*_0_ is the concentration of the tested element digested by the UAEO method without standard solution, while *C*_1_ is that by standard solution. *C*_spk_ represents the theoretical concentration of spiked elements.

### 2.5. Chlorine Quantitative Analysis

The digestion solution was taken out from the digestion cuvette in 0, 5, 10, 15, 20, 25, 30, 35, 40, and 45 min during the UAEO digestion method. Every sample was immediately placed in the ice-water bath to cool down, diluted, and filtered to be tested.

The concentration of chlorine was measured according to the *N*,*N*-diethyl-*p*-phenylenediamine (DPD) colorimetric method [27] using a UV 2600 spectrophotometer (Shimadzu, Kyoto, Japan).

### 2.6. Particle Size Distribution and Micromorphology

The particle size distribution of the digestion sample was measured using a Mastersizer 3000 laser particle size analyzer (Malvern Panalytical. Ltd., Cambridge, UK). The microtopographic analysis of the insoluble solid in the digestion sample was carried out using a field-emission scanning electron microscope (SEM) (JSM-IT300LV, Jeol, Japan, operating at 20 kV) and an optical microscope (OM) (BX51, Olympus, Tokyo, Japan).

### 2.7. Statistical Analysis

All statistical analyses were performed using PASW Statistics 18 software (SPSS Inc., Armonk, NY, USA) with analysis of variance (ANOVA) and Dunnet’s test [28].

## 3. Results

### 3.1. Effects of UAEO Operating Parameters on Digestion Efficiency

**Figure 3:**
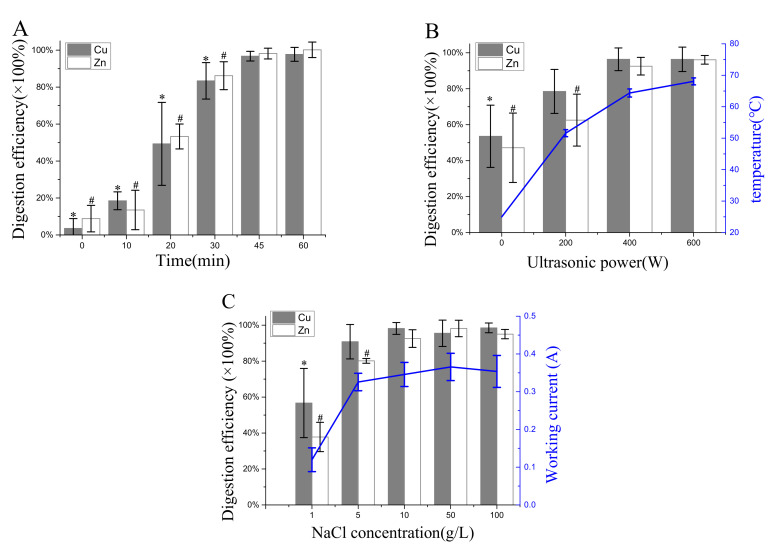
Variations in Cu and Zn digestion efficiency, solution temperature, and working current during UAEO digestion method. (**A**) Cu and Zn digestion efficiency was tested for different periods at 400 W ultrasonic power with a NaCl concentration of 10 g/L. Significant differences between each digestion time and 60 min group were found with the Dunnet’s test; * *p* < 0.05 for Cu, ^#^ *p* < 0.05 for Zn. (**B**) Digestion efficiency was tested at 0, 200, 400, and 600 W ultrasonic power for 45 min with a NaCl concentration of 10 g/L, and the solute temperature was detected. Significant differences between each ultrasonic power and 600 W group were found with the Dunnet’s test; * *p* < 0.05 for Cu, ^#^ *p* < 0.05 for Zn. (**C**) Digestion efficiency was tested with NaCl concentrations of 1, 5, 10, 50, and 100 g/L at 400 W ultrasonic power for 45 min, and the working current was tested using an electronic working station. Significant differences between each NaCl concentration and 100 g/L group were found with Dunnet’s test; * *p* < 0.05 for Cu, ^#^ *p* < 0.05 for Zn.

As shown in Figure 3, the digestion efficiency was positively related to the digestion time, ultrasonic frequency, and NaCl concentration. Further, Cu and Zn digestion efficiency was tested for different periods at 400 W ultrasonic power with a NaCl concentration of 10 g/L (Figure 3A). The digestion efficiency increased rapidly in the first 30 min and then reached a plateau in 30–60 min. The Dunnet’s test results showed no significant difference (*p* > 0.05) in digestion efficiency between 45 and 60 min. As shown in Figure 3B, the digestion efficiency increased with the increase in ultrasonic power with 45 min digestion time and 10 g/L NaCl concentration; the solute temperature was raised at the same time. The digestion efficiency had no significant difference between 400 W and 600 W ultrasonic power, although the solute temperature slightly increased. Finally, the optimal concentration of oxidation substrate NaCl was tested from 1 to 100 g/L with 45 min digestion time and 400 W ultrasonic power, as shown in Figure 3C. The results showed that an increase in NaCl concentration led to more working current and higher digestion efficiency until the NaCl concentration exceeded 10 g/L. With the optimal UAEO operating parameters (digestion time: 45 min; ultrasonic power: 400 W; NaCl concentration: 10 g/L), Cu and Zn digestion efficiency was (96.8 ± 2.6) and (98.5 ± 2.9)%, respectively.

### 3.2. UAEO Digestion Recovery Rate

**Figure 4:**
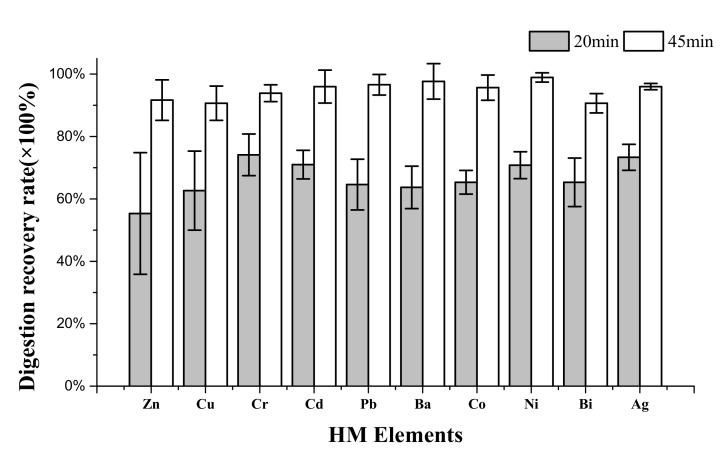
UAEO standard digestion rate of 10 primary HMs in dairy slurry.

The digestion recovery rate experiments were operated with spiked samples to evaluate the UAEO digestion effect for trace HMs. The 20 min and 45 min standard digestion rate for 10 HMs was tested, and the results are shown in Figure 4. After a 20 min UAEO digestion operation, the recovery rate of all 10 HMs exceeded 70%. When the processing duration further reached 45 min, all digestion recovery rates exceeded 90%. The specific results are listed in Table 1.

### 3.3. Free Chlorine

**Figure 5:**
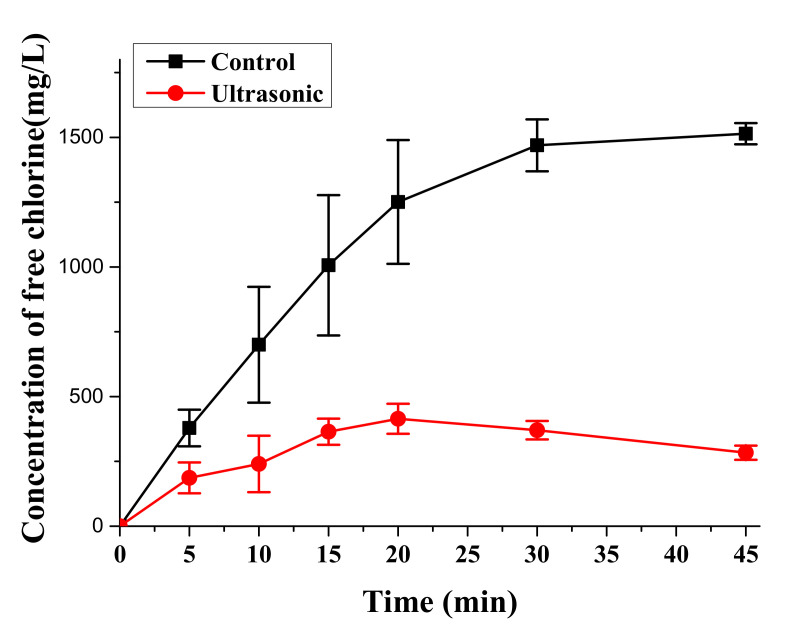
Variations in the concentration of free chlorine during digestion operation (45 min).

The concentration of free chlorine was tested by the DPD method to analyze the level of oxidation medium in the digestion solution. As shown in Figure 5, the ultrasound intervention would reduce the free chlorine increment speed in the reaction system. Specifically, when the ultrasound function module was turned off, the free chlorine concentration was maintained at about 1500 mg/L after digestion for 30 min. In another case, with ultrasound intervention, the free chlorine concentration could only reach the maximum (414 mg/L) at 20 min and, further, slightly decreased to 283 mg/L in 45 min instead of continuing to increase.

### 3.4. Effect of the UAEO Process on Particle Size Distribution and Micromorphology

**Figure 6:**
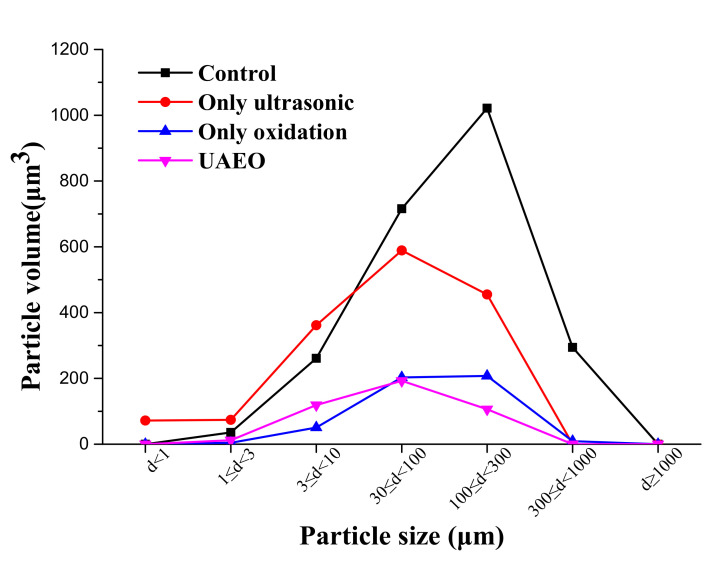
Particle size distribution of undissolved solid matters in the dairy slurry sample after different digestion treatments (45 min).

As shown in Figure 6, UAEO digestion treatment indeed changed the undissolved particle size distribution. Before digestion, the particles with a diameter between 100 and 300 μm contributed to the main particle volume (1021 μm^3^). Under the influence of oxidation, the total particle volume sharply declined, although the size distribution did not show an obvious change. Under the influence of ultrasound intervention, the size distribution moved toward small sizes, and the total particle volume had an obvious decline as well. When the UAEO digestion method worked normally, the effect on the change in particle size was comparable to a coupling of ultrasound and oxidation; the greatest abundance of particle size was changed to a smaller diameter range between 30 and 100 μm, which had a much smaller particle volume (193 μm^3^) compared with the untreated sample.

**Figure 7:**
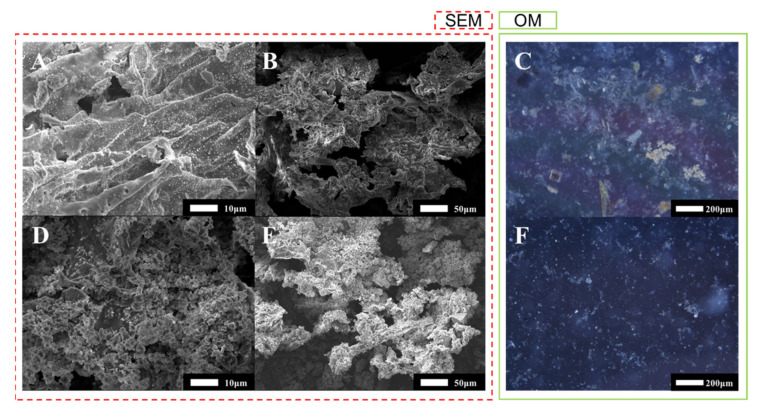
FE-SEM and OM images of undissolved solid matters before (**A**–**C**) and after (**D**–**F**) UAEO digestion.

As shown in Figure 7, the micromorphology of undissolved solid matters before and after UAEO digestion was observed using FE-SEM and OM. The undissolved solid matters before digestion (Figure 7A,B) presented typical micromorphological characteristics of the dairy slurry, an irregular blocky structure. As shown in Figure 7D,E, the blocky structure appeared as arc edge and cellular structure after the UAEO digestion method. More macroscopic OM images (Figure 7C–E) showed that the digestion process not only reduced the amount of undissolved solid matters but also made them fragmented and blanched.

## 4. Discussion

### 4.1. Digestion Theory of the UAEO Method

The constitution of UAEO technology is shown in Figure 1. The digestion device comprised two open cuvettes of NaCl solution, which was connected with a NaCl salt bridge. Under the influence of electric potential difference, negative ions moved toward the working electrode and positive ions moved toward the auxiliary electrode. The reactions in the digestion cuvette were expressed by Equations (3)–(6). Hydroxyl radicals and free chlorine were generated in the digestion process, and redundant H^+^ provided an acidic environment for easy distribution of HMs. In addition, the ultrasound function module produced ultrasonic waves, generated shock waves to break large particles and solids, accelerated the redox reaction, and then improved the digestion effect [19,29]. By setting a classic three-electrode system and ultrasound function module, the synergy between ultrasonic effect and electrochemical digestion was ensured in the digestion process to improve the digestion effect.

(Ru–Ir)@Ti + H_2_O→(Ru–Ir)@Ti(OH) + H^+^ + e^−^ (3)

Cl^−^ + OH→·ClO^−^ + H^+^ + e^−^ (4)

2Cl^−^→Cl_2_ + 2e^−^ (5)

Cl_2_ + 2H_2_O→HClO/ClO^−^ + Cl^−^ + H^+^/2H^+^ (6)

### 4.2. Digestion Efficiency of the UAEO Method

A previous investigation [12] indicated that the concentrations of Zn and Cu were significantly higher than those of the other HMs in the dairy farm slurry. Therefore, a series of digestion efficiency experiments were designed to explore the optimal operating parameters of the UAEO apparatus based on the results of Zn and Cu digestion efficiency. Figure 3A shows the effect of digestion time on digestion efficiency at 400 W ultrasonic power with a NaCl concentration of 10 g/L. As expected, the digestion efficiency increased continually in the first 45 min due to sustaining electrochemical oxidation and ultrasound function. As shown in Figure 3B, the digestion efficiency was positively associated with ultrasonic power. The ultrasound intervention could not only increase the temperature of the digestion solution to accelerate the redox reaction but generate shock waves to break large particles and solids. When the ultrasonic power was kept at 400 W or above, the solution temperature reached 60 °C and the digestion efficiency reached the maximum. NaCl was used as an oxidation substrate to generate an oxidizing agent and working current. Figure 3C shows that a low concentration of NaCl restricted the oxidation reaction rate. However, excessively high salinity possibly had a negative impact on the follow-up analysis. Taken together, 10 g/L NaCl was the ideal concentration for the UAEO digestion method. The foregoing conclusions not only proved that the UAEO digestion method could sufficiently release Zn and Cu, which were the main HMs in the dairy slurry, but also found the optimal UAEO operating parameters (digestion time: 45 min; ultrasonic power: 400 W; NaCl concentration: 10 g/L).

An HM recovery rate test was carried out to verify the UAEO digestion effect on broad-spectrum HMs. The element form in spiked digestion samples was different from that in normal samples because the spiked process could form nonionic compounds only in solution and not in insoluble particulate matter. The results of the recovery rate could still partly prove the applicability of UAEO digestion for different HMs. As shown in Figure 4, the UAEO digestion method demonstrated adequate ability for 10 primary HM elements, illustrating that the UAEO method had indiscriminate digestion function for dairy slurry HMs.

### 4.3. Digestion Mechanism of the UAEO Method

Free chlorine was the main oxidation medium in the digestion solution, according to Equations (3)–(6). Therefore, the variations in the concentration of free chorine were tested to discuss the key mechanism of the UAEO method. As shown in Figure 5, the variation curves demonstrated a high concentration of free chorine without ultrasound intervention. Due to the great oxidizability of free chlorine, it was easy to degrade organics and release HMs oxidatively. Although the ultrasound intervention decreased the maximum concentration of free chorine due to vibration and temperature effect, 414 mg/L free chorine still theoretically had sufficient oxidative ability. On the contrary, the inhalation toxicology of excessive chlorine might influence the health of operators [30]. Because the dairy slurry had many undissolved particles, single electrochemical oxidation digestion could not process them. The ultrasound intervention could exactly constitute this function attributed to its crushing capacity. These hypotheses were verified by the results of particle size distribution analysis. As shown in Figure 6, the particle size distribution obviously moved toward small size, although the ultrasound intervention could not sharply decrease the total volume of particles. Moreover, under the oxidation effect, the total volume and the proportion of bulky particles obviously declined. To intuitively demonstrate the change in particle form during the digestion process, the micromorphology results are shown in Figure 7. The ultrasound intervention led to the fragmentation of the bulky particles, and electrochemical oxidation made an irregular blocky structure form arc edges and a cellular structure.

### 4.4. Advantages of the UAEO Digestion Method

The conventional digestion methods usually required strong acids as digestion reagents, and the digestion process was completed under high-temperature and high-pressure conditions, which depended on large-scale digestion equipment. They were difficult to meet the requirements of the onsite test. UAEO was a novel and efficient method for dairy farm slurry digestion. The electrochemical oxidation process provided a sufficient oxidation agent. The ultrasound intervention not only physically broke the particles and solids in the dairy slurry but also catalyzed the improvement in oxidation digestion efficiency. On the other hand, the UAEO digestion method is an economical approach owing to its low-cost reagent and simple apparatus design. The UAEO digestion method was fast and convenient and ensured digestion efficiency, thus having a good application prospect.

## 5. Conclusions

Dairy farm slurry is an important cheap biomass resource rich in mineral nutrients and lignocellulose. However, the abuse of feed and the lack of harmless treatment lead to HM pollution. An innovative digestion method was established in this study, which combined digestion efficiency and process convenience. According to the experimental results, the UAEO method can sufficiently digest all main HMs (Zn and Cu) or trace HMs in slurry. This might be further beneficial to agriculture biomass resource quality control and effective transformation.

## Figures and Tables

**Table 1:** Concentrations and recovery rates (%) in the spiked samples digested by the UAEO method.

Element	Certified Values (mg/L)	Found Values (mg/L) and Recovery Rates (%)			
		20 min		45 min	
Zn	0 ^a^	1.25 ± 0.28	55.3%	2.24 ± 0.45	91.7%
	1 ^b^	1.8 ± 0.09		3.16 ± 0.44	
Cu	0	0.29 ± 0.05	62.7%	0.54 ± 0.07	90.7%
	1	0.92 ± 0.17		1.45 ± 0.09	
Cr	0	0.06 ± 0.03	74.1%	0.11 ± 0.03	93.9%
	5	3.77 ± 0.35		4.81 ± 0.16	
Cd	0	ND ^c^	71%	ND	96%
	1	0.71 ± 0.05		0.96 ± 0.05	
Pb	0	ND	64.6%	0.08 ± 0.04	96.6%
	10	6.46 ± 0.81		9.74 ± 0.36	
Ba	0	ND	63.7%	ND	97.7%
	1	0.64 ± 0.07		0.98 ± 0.06	
Co	0	ND	65.3%	ND	95.7%
	1	0.65 ± 0.04		0.96 ± 0.04	
Ni	0	ND	70.8%	ND	98.9%
	5	3.54 ± 0.22		4.95 ± 0.08	
Bi	0	ND	65.3%	ND	97.8%
	10	6.53 ± 0.78		9.78 ± 0.31	
Ag	0	ND	73.3%	ND	96%
	1	0.73 ± 0.04		0.96 ± 0.01	

^a^ Unspiked sample; ^b^ Sample spiked with multi-element standard solution; ^c^ Not detected and calculated as 0.

## Data Availability

The raw/processed data required to reproduce these findings cannot be shared at this time as the data also forms part of an ongoing study.

## Supplementary Materials

The following are available online at https://www.mdpi.com/article/10.3390/ma14164562/s1. Table S1, Operating program of the microwave apparatus. Table S2, ICP-OES instrumental parameters for the analysis of 10 objective HMs.

## References

[1] Lu Y.Z., Zhuo C., Li Y.J., Li H.S., Yang M.Y., Xu D.N., He H.Z. (2020). Evaluation of filamentous heterocystous cyanobacteria for integrated pig-farm biogas slurry treatment and bioenergy production. Bioresour. Technol..

[2] Muhammad L., Tareq A. (2021). A novel solution towards zero waste in dairy farms: A thermodynamic study of an integrated polygeneration approach. Energy Convers. Manag..

[3] Tethi B., Shashi B., Kumar P.S., Shaon R.C. (2021). An eco-friendly strategy for dairy wastewater remediation with high lipid microalgae-bacterial biomass production. J. Environ. Manag..

[4] Wang X., Ledgard S., Luo J., Guo Y., Zhao Z., Guo L., Liu S., Zhang N., Duan X., Ma L. (2018). Environmental impacts and resource use of milk production on the North China Plain, based on life cycle assessment. Sci. Total Environ..

[5] Zhu D., Wei Y., Zhao Y., Wang Q., Han J. (2018). Heavy Metal Pollution and Ecological Risk Assessment of the Agriculture Soil in Xunyang Mining Area, Shaanxi Province, Northwestern China. Bull. Environ. Contam. Toxicol..

[6] Jean H., Eléonore L., Carole S., Arnaud H. (2021). Identifying the resource use and circularity in farm systems: Focus on the energy analysis of agroecosystems. Resour. Conserv. Recycl..

[7] Li J., Akdeniz N., Kim H.H.M., Gates R.S., Wang X., Wang K. (2021). Optimal manure utilization chain for distributed animal farms: Model development and a case study from Hangzhou, China. Agric. Syst..

[8] Phitsanu T., Alongkot B., Suwicha K., Srisamai W., Juree P., Suwanna T., Hathairad H., Ratchaneekorn M., Ramnaree N., Sutha K. (2011). Comparative study of heavy metal and pathogenic bacterial contamination in sludge and manure in biogas and non-biogas swine farms. J. Environ. Sci..

[9] Roa Engel Carol A., Van Gulik Walter M., Leonie M., Van Der Wielen Luuk A.M., Straathof Adrie J.J. (2011). Development of a low pH fermentation strategy for fumaric acid production by Rhizopus oryzae. Enzym. Microb. Technol..

[10] Wen Z., Liao W., Chen S. (2005). Production of cellulase by Trichoderma reesei from dairy manure. Bioresour. Technol..

[11] Li H., Dai M., Dai S., Dong X. (2018). Current status and environment impact of direct straw return in China’s cropland–A review. Ecotoxicol. Environ. Saf..

[12] Liu W.-R., Zeng D., She L., Su W.-X., He D.-C., Wu G.-Y., Ma X.-R., Jiang S., Jiang C.-H., Ying G.-G. (2020). Comparisons of pollution characteristics, emission situations, and mass loads for heavy metals in the manures of different livestock and poultry in China. Sci. Total Environ..

[13] Lin Z., Chen X., Xi Z., Lin S., Sun X., Jiang X., Tian H. (2018). Individual heavy metal exposure and birth outcomes in Shenqiu county along the Huai River Basin in China. Toxicol. Res..

[14] Xiaodong W., Yu Z., Qingwen D., Shoulian J., Xia Z., Jie G. (2012). Investigation of novel rapidly synergistic cloud point extraction pattern for bismuth in water and geological samples coupling with flame atomic absorption spectrometry determination. Spectrochim. Acta Part A Mol. Biomol. Spectrosc..

[15] Chen L., Lei Z., Yang S., Wen X. (2017). Application of portable tungsten coil electrothermal atomic absorption spectrometer for the determination of trace cobalt after ultrasound-assisted rapidly synergistic cloud point extraction. Microchem. J..

[16] Wen X., Yang S., Zhang H., Deng Q. (2016). Combination of knotted reactor with portable tungsten coil electrothermal atomic absorption spectrometer for on-line determination of trace cadmium. Microchem. J..

[17] Wang Z., Sun X., Li C., He X., Liu G. (2016). On-site detection of heavy metals in agriculture land by a disposable sensor based virtual instrument. Comput. Electron. Agric..

[18] Ajay P.V.S., Printo J., Kiruba D.S.C.G., Susithra L., Takatoshi K., Sivakumar M. (2017). Colorimetric sensors for rapid detection of various analytes. Mater. Sci. Eng. C.

[19] Santos Daniele C.M.B., Carvalho Larissa S.B., Lima Daniel C., Leão Danilo J., Teixeira Leonardo S.G., Korn Maria Graças A. (2014). Determination of micronutrient minerals in coconut milk by ICP OES after ultrasound-assisted extraction procedure. J. Food Compos. Anal..

[20] Luisa A.M., Elisabetta M., Carmela P., Matteo V., Elisa S., Paola M., Silvia C. (2018). Optimization and validation of a fast digestion method for the determination of major and trace elements in breast milk by ICP-MS. Anal. Chim. Acta.

[21] Fernandes D.O.A., Santos C., Rossana B.S., Araujo N.A.R. (2018). The use of diluted formic acid in sample preparation for macro- and microelements determination in foodstuff samples using ICP OES. J. Food Compos. Anal..

[22] Krishna M.V.B., Chandrasekaran K., Venkateswarlu G., Karunasagar D. (2019). Development of a simple and rapid microwave-assisted extraction method using very dilute solutions of perchloric acid and hydrogen peroxide for the multi-elemental analysis of food materials by ICP-OES: A green analytical method. Microchem. J..

[23] Kuznetsova O.V., Burmii Z.P., Orlova T.V., Sevastyanov V.S., Timerbaev A.R. (2019). Quantification of the diagenesis-designating metals in sediments by ICP-MS: Comparison of different sample preparation methods. Talanta.

[24] Rovasi A.F., Cícero D.N.P., Camera L.G., Denise B., Carine V., Machado L. (2020). Simultaneous determination of Fe and Ni in guarana (Paullinia cupana Kunth) by HR-CS GF AAS: Comparison of direct solid analysis and wet acid digestion procedures. J. Food Compos. Anal..

[25] Da Silva I.J.S., Lavorante André F., Paim A.P.S., Da Silva M.J. (2020). Microwave-assisted digestion employing diluted nitric acid for mineral determination in rice by ICP OES. Food Chem..

[26] Vimlesh C., Surendra P. (2013). ICP-OES assessment of heavy metal contamination in tropical marine sediments: A comparative study of two digestion techniques. Microchem. J..

[27] Rice E.W., Baird R.B., Eaton A.D., Clesceri L.S. (2012). Standard Methods for the Examination of Water and Wastewater.

[28] Ge B., Hunter J., Hunter W. (2005). Satistics for Experimenters: Design, Innovation, and Discovery.

[29] Tadeo J.L., Sánchez-Brunete C., Albero B., García-Valcárcel A.I. (2010). Application of ultrasound-assisted extraction to the determination of contaminants in food and soil samples. J. Chromatogr. A.

[30] Musah S., Schlueter C.F., Humphrey D.M., Powell K.S., Roberts A.M., Hoyle G.W. (2017). Acute lung injury and persistent small airway disease in a rabbit model of chlorine inhalation. Toxicol. Appl. Pharmacol..

