# Supplementary material for: An Innovative Digestion Method: Ultrasound-Assisted Electrochemical Oxidation for the Onsite Extraction of Heavy Metal Elements in Dairy Farm Slurry
Source: Materials (Basel). 2021 Aug 13;14(16):4562. doi: 10.3390/ma14164562 (PMC8400106; doi:10.3390/ma14164562)
Supplement: Supplementary file 1 [file materials-14-04562-s001.zip › materials-1276999-supplementary.pdf]

Table S1. Operating program of microwave apparatus.

Step	Temperature (°C)	Hold Time (min)	Pressure Limit (atm)
1	80	5	5
2	120	5	20
3	180	20	30

Table S2. ICP-OES instrumental parameters for the analysis of 10 objective HMs.

Parameter	Value	Parameter	Value
Clean pump speed	50 rpm	Nebulizer gas flow	0.50 L/min
Analysis pump speed	50 rpm	Calibration type	Zero intercept, linear
RF power	1150 W	Replicates	3
Plasma gas flow	12 L/min	Carrier solution	1% HNO_3
Auxiliary gas flow	0.50 L/min	Rinse solution	5% HNO_3
